# Bennett fracture combined with hamate fracture: carpometacarpal joint ‘diagonal’ fracture and dislocation: a case report

**DOI:** 10.1186/s12891-023-06588-3

**Published:** 2023-06-10

**Authors:** Wei-Bin Wu, Yun-Feng Du, Hong-Xing Wang, Feng Liang

**Affiliations:** Department of Orthopaedics, Jiaozuo Coal Industry (Group) Co. Ltd. Central Hospital, Jiaozuo, Henan 454000 P.R. China

**Keywords:** Bennett fracture, Carpometacarpal joint, Fracture dislocation, Hamate bone

## Abstract

**Background:**

Multiple carpometacarpal fractures and dislocations are rare. This case report describes a novel multiple carpometacarpal injury, namely, ‘diagonal’ carpometacarpal joint fracture and dislocation.

**Case presentation:**

A 39-year-old male general worker sustained a compression injury to his right hand in the dorsiflexion position. Radiography indicated a Bennett fracture, hamate fracture, and fracture at the base of the second metacarpal. Subsequent computed tomography and intraoperative examination confirmed an injury to the first to fourth carpometacarpal joint along a diagonal line. The normal anatomy of the patient’s hand was successfully restored via open reduction combined with Kirschner wire and steel plate fixation.

**Conclusion:**

Our findings highlight the importance of taking the injury mechanism into account to avoid a missed diagnosis and to choose the best treatment approach. This is the first case of ‘diagonal’ carpometacarpal joint fracture and dislocation to be reported in the literature.

## Background

Multiple carpometacarpal (CMC) fractures and dislocations are rare, occur in less than 1% of hand injuries, and are usually caused by high-energy trauma. Up to 70% of CMC fractures and dislocations are either overlooked or misdiagnosed [1]. Missed diagnosis or delayed treatment often leads to wrist pain, reduced grip strength, degenerative arthritis, and an inability to return to work [2].

In the literature, CMC fractures are mainly reported as single-joint injuries and rarely as dislocations of the second to fifth CMC joints or all five CMC joints [3, 4]. Furthermore, whether CMC injury is better treated with closed or open reduction or Kirschner wire or plate fixation remains controversial. In this case report, we describe a ‘diagonal’ CMC joint fracture and dislocation including Bennett fracture, hamate fracture, fracture at the base of the second metacarpal, and dislocation of the first to fourth CMC joints. To the best of our knowledge, this is the first case of ‘diagonal’ CMC joint fracture and dislocation to be reported in the literature.

## Case presentation

A 39-year-old male general worker sustained a compression injury to his right hand in the dorsiflexion position while pushing a heavy object that was rolling down. Physical examination of the injury revealed noticeable swelling of the entire hand, palpable high skin tension, numbness in each finger, and a positive Tinel sign at the wrist; the pain was elicited on palpating the CMC joint. Owing to the swelling and pain, active mobilisation of the metacarpophalangeal and interphalangeal joints was limited. The mobility of the wrist joint was normal, and the blood circulation in the fingertips was good.

**Fig. 1:**
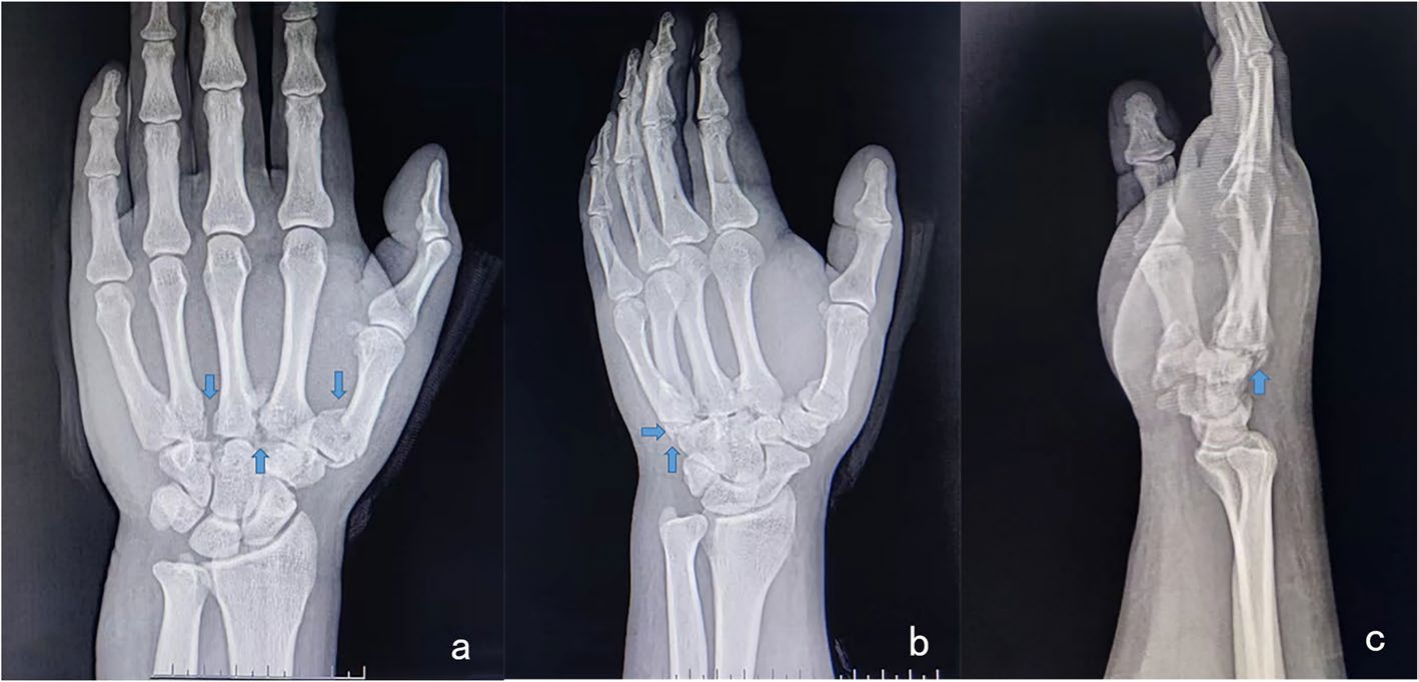
Preoperative radiographic findings. **a** The anteroposterior radiograph shows fracture and dislocation of the bases of the first and second metacarpals and enlargement of the spaces at the bases of the third and fourth metacarpals. **b** The oblique radiograph shows dorsal displacement of the base of the fourth metacarpal with compressed fracture fragments of the hamate bone. **c** The lateral radiograph shows indistinguishable dorsal dislocation of the carpometacarpal joint

We considered the CMC joint space as a line, the metacarpal base was below the line, the distal carpal bones were on the line, and the middle finger and the capitate bone were the centre line. The angle between the base of the first metacarpal bone and the hamate bone was considered the diagonal angle. Radiography of the right hand revealed fracture and dislocation of the bases of the first and second metacarpals and enlarged spaces at the bases of the third and fourth metacarpals (anteroposterior radiograph, Fig. 1a); dorsal displacement of the base of the fourth metacarpal with compressed fracture fragments of the hamate bone (oblique radiograph, Fig. 1b); and indistinguishable dorsal dislocation of the CMC joint (lateral radiograph, Fig. 1c).

**Fig. 2:**
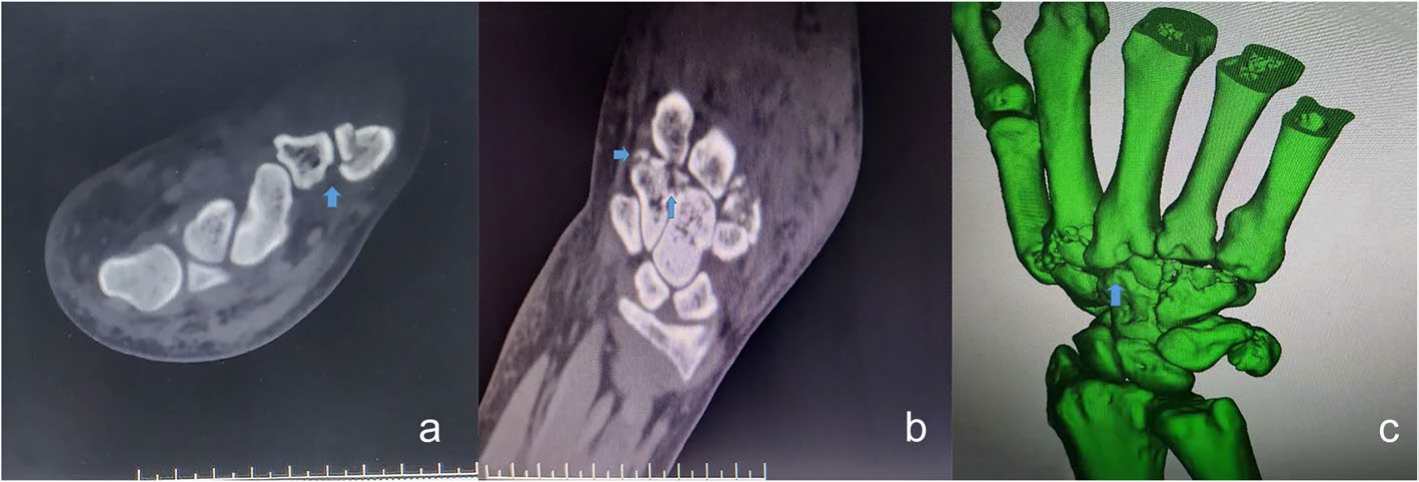
Preoperative computed tomography (CT) findings. **a** Cross-section CT reveals a Bennett fracture. **b** Coronal CT shows a fracture at the base of the second metacarpal and hamate bone. **c** Three-dimensional CT reveals dislocation of the third carpometacarpal joint

Based on the injury mechanism and physical examination results, injury to the third CMC joint or even the fifth CMC joint was suspected. Because the aforementioned radiographic examination could not detect all injuries, computed tomography (CT) was performed. CT confirmed the dorsal dislocation of the third CMC joint (Fig. 2). The fifth CMC joint seemed normal, which was confirmed intraoperatively.

The patient underwent open reduction and internal fixation under brachial plexus anaesthesia 10 days later. After the Bennett fracture was reduced and clamped, two Kirschner wires were used to fix the fractured end from the radial side of the distal end of the metacarpal fracture to the trapezium bone. The fracture of the base of the second metacarpal was comminuted; however, there was no bone mass loss. Therefore, the second CMC joint fracture dislocation was reduced before being obliquely and longitudinally fixed to the capitate bone with a Kirschner wire.

Intraoperative exploration revealed that the third CMC joint was dislocated and significantly unstable, and the joint capsule was ruptured. Therefore, a Kirschner wire was used for oblique and longitudinal fixation, which was conducive to the healing of the joint capsule. The compression fracture of the hamate bone was comminuted, with bone mass loss. Transarticular fixation was performed with miniature steel plates. The compressed fracture fragments and articular surface were restored to their original positions, and one Kirschner wire was added to strengthen the fixation. The median nerve was not examined because the patient’s symptoms had been significantly relieved before surgery and were considered to be caused by traction injury.

**Fig. 3:**
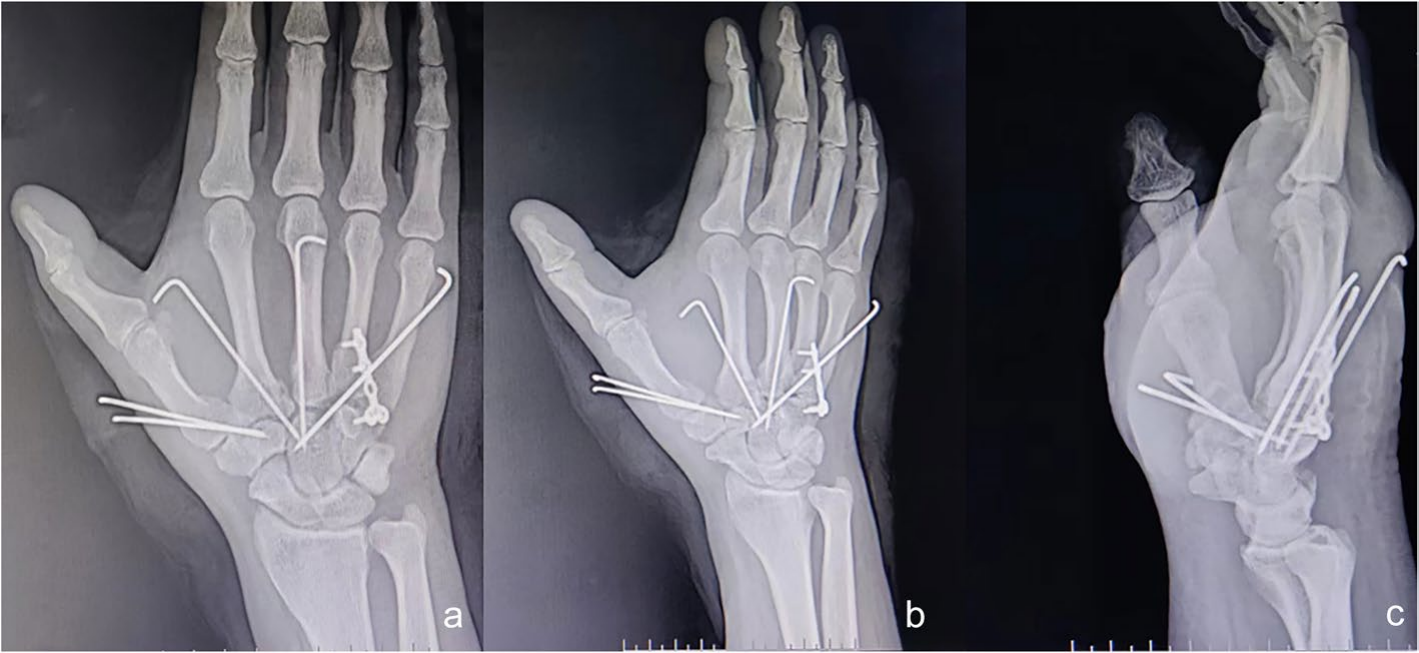
Postoperative radiography findings. The radiographs in the anteroposterior (**a**), oblique (**b**), and lateral (**c**) positions show good alignment of the fracture and dislocation; the anatomic bony relationships have been restored

**Fig. 4:**
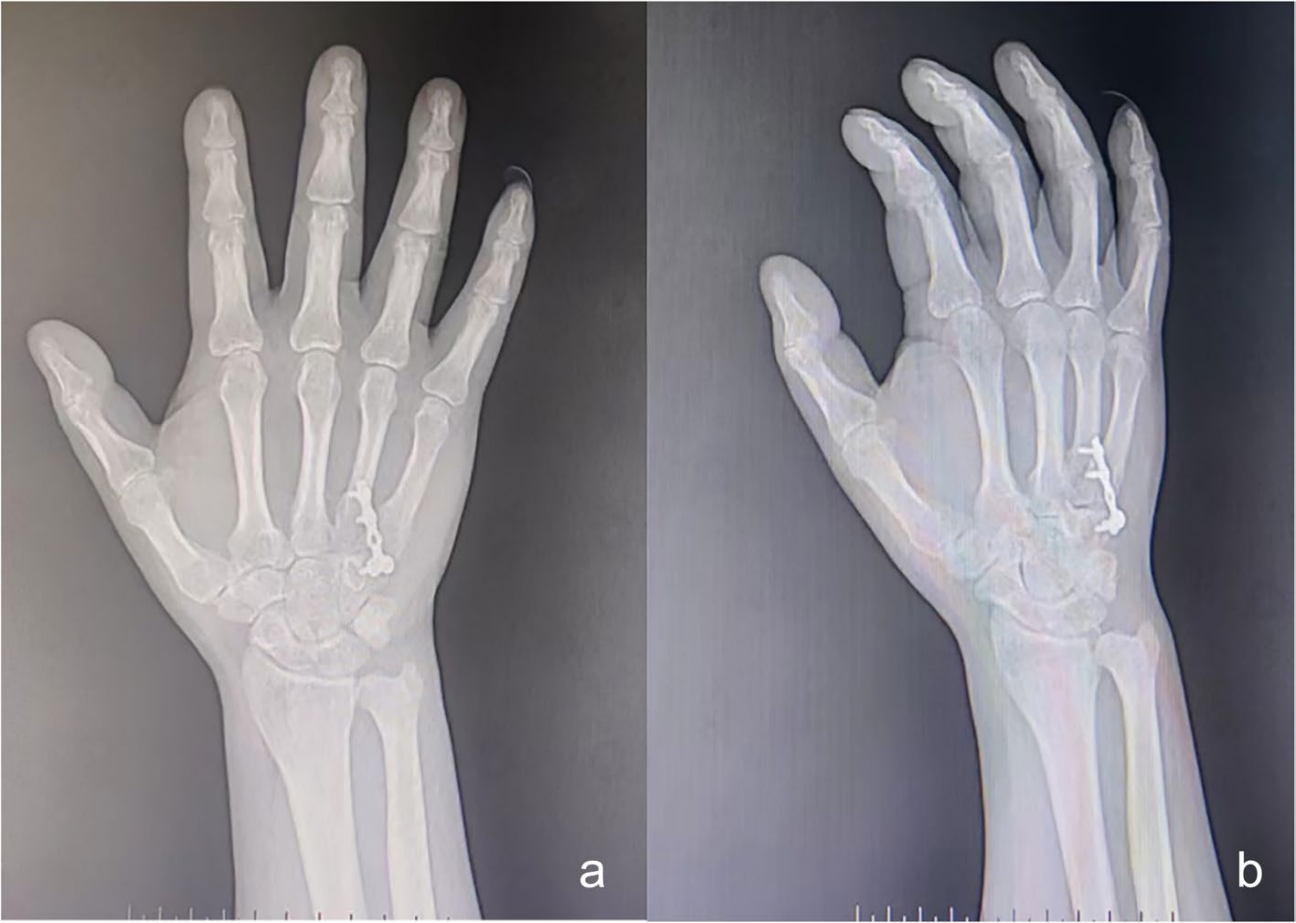
Healing after surgery. Four months after surgery, radiographs of the hand in the anteroposterior (**a**) and oblique (**b**) positions before removal of the steel plate show favourable healing of the fracture and no dislocation of the carpometacarpal joints

**Fig. 5:**
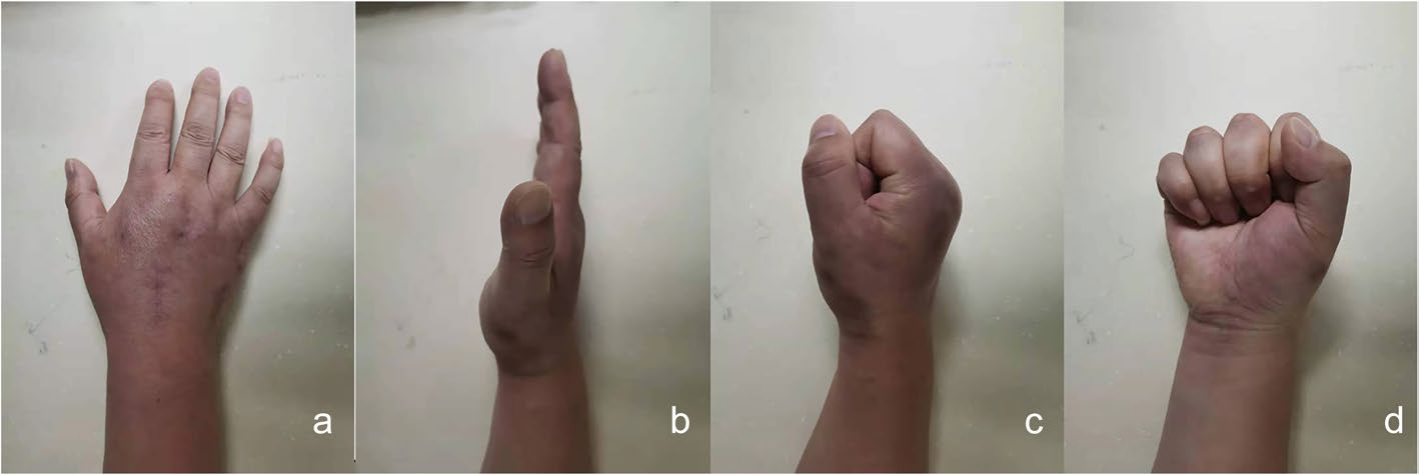
Hand movements after surgery. The appearance of the injured hand (**a**), the opening of the palm (**b**), and making a fist (**c**, **d**) 5 months after surgery

Postoperative radiography indicated favourable alignment of each CMC joint (Fig. 3). The patient was advised to exercise each metacarpophalangeal and interphalangeal joint with plaster cast protection for 3 weeks after surgery. At that time, the cast was removed, and functional exercises of the wrist were started. The Kirschner wire was pulled out 6 weeks after the surgery. Whereas the plates were not removed because they did not create any negative impact on the patient’s range of motion (flexion and extension). Subsequently, functional exercises of the first CMC joint were initiated. Radiographs obtained 4 months after surgery revealed favourable fracture healing (Fig. 4), and the symptoms associated with the median nerve were completely relieved. The patient regained mobility of the injured hand 5 months after surgery (Fig. 5) and returned to work 7 months after surgery. During the follow-up period of 1 year and 4 months, there was no pain in the injured hand, and the grip strength, muscle strength, and joint range of motion of the operated hand were comparable to those of the contralateral hand.

## Discussion and conclusions

The CMC joints are mainly connected and maintained by ligaments, as well as by bony and capsular structures. By dissecting 10 fresh specimens, Dzwierzynski et al. [5] demonstrated that four groups of ligaments in each CMC joint enhance the stability of its joint capsule. Among these groups, the intermetacarpal ligaments are the strongest, followed in order by the dorsal ligaments and volar ligaments. Nanno et al. [6] also analysed the dorsal, volar, intermetacarpal, and other thick ligaments and found them firmly fixed to the carpal bone. Therefore, CMC dislocation, especially multiple CMC dislocation, is extremely rare [6].

The CMC joint is generally spared from injury because of its strong and stable structure. However, a relatively strong indirect exerted force can injure the CMC joint, and the injury often involves intra-articular fractures accompanied by joint instability. Force exerted on multiple fingers can result in fractures and dislocations of multiple CMC joints; however, this is rare in clinical practice and easy to overlook. The base of the third metacarpal is connected to the bases of the second and fourth metacarpals; thus, injury to the base of the third may increase the risk of trauma to the bases of the second and fourth and vice versa. Consequently, physicians should be vigilant so that all fractures and dislocations are identified [7].

The injury mechanism, in this case, was trauma to the hand on the palmar side, causing injury in the dorsiflexion position of the palm. Indirect force resulted in Bennett fracture and dislocation, fracture and dislocation of the base of the second metacarpal, compression fracture of the hamate bone, and dislocation of the fourth CMC joint. With the bilateral columns being injured, can the middle column be spared? In our study, the mechanism of injury suggested dislocation of the third CMC joint, which was confirmed via CT and intraoperative exploration. If the patient’s medical history is not thoroughly recorded and physical examination is not carefully performed, CMC fractures and dislocations are likely to be missed, potentially resulting in adverse consequences, such as pain, arthritis, and reoperation.

Treatment of multiple CMC joints via closed reduction combined with plaster cast fixation often leads to reduction loss and poor recovery. Open reduction may be preferable to closed reduction because in closed reduction, retention of fracture fragments and soft tissue interposition impede anatomical reduction, causing arthritis [7–9]. The risk of re-dislocation is higher after conservative treatment of CMC dislocation than after open reduction, especially in patients with intra-articular metacarpal and carpal fractures [10, 11].

Steel plate fixation and Kirschner wire fixation are the most commonly used fixation methods. Kirschner wire fixation is reportedly superior to steel plate fixation because of its high elasticity, which reduces CMC rigidity to the greatest extent possible [12].

We have considerable experience with Bennet fractures, and although several treatment options are available, we prefer open reduction because it allows reduction of the articular surface under direct vision; as such, it prevents the subsequent onset of arthritis caused by intra-articular steps and gaps and produces favourable outcomes. In treating hamate and metacarpal fractures and subluxations, Kirschner wire fixation is not sufficiently firm to provide early stability and is even less useful for achieving early functional movements of the hand [13]. Moreover, because the deep branch of the ulnar nerve is close to the volar aspect of the hamate bone, it can easily be injured while applying or removing the Kirschner wire [14]. The clinical efficacy of a dorsal supporting plate in the treatment of fourth and fifth CMC joint fractures and dislocations is satisfactory [15]. Elastic fixation is applied when the CMC joints can be stabilised, whereas rigid fixation is required in cases of compression, bone mass loss, or joint instability. We used transarticular miniature steel plates and Kirschner wires owing to compression and comminuted fracture of the hamate bone; this approach was effective in the later stage. However, our study has a limitation. We did not record the exact range of motion of the operated hand compared to the contralateral hand.

In summary, we reported a case in which the diagnosis could have been easily missed. In analysing the patient’s condition and potential treatments, based on our study, we make the following suggestions: 1. Preoperatively, medical history should be thoroughly inquired, and physical and radiological examinations should be carefully performed to prevent a missed diagnosis. Attention should especially be paid to the mechanism of injury and the anatomical relationship. 2. In cases of nerve injury, preoperative relief of symptoms and the necessity of surgical exploration should be carefully confirmed. 3. In cases of intra-articular fractures (hamate compression fracture in this case), trans-metacarpophalangeal joint fixation with a locked plate should be applied to increase stability and enable early mobility of the affected structures.

## Data Availability

The datasets used and/or analysed during the current study are available from the corresponding author on reasonable request.

## Abbreviations

CMC: Carpometacarpal
CT: Computed tomography
